# Eating Competence among a Select Sample of Brazilian Adults: Translation and Reproducibility Analyses of the Satter Eating Competence Inventory

**DOI:** 10.3390/nu12072145

**Published:** 2020-07-19

**Authors:** Fabiana Lopes Nalon de Queiroz, Eduardo Yoshio Nakano, Verônica Cortez Ginani, Raquel Braz Assunção Botelho, Wilma Maria Coelho Araújo, Renata Puppin Zandonadi

**Affiliations:** 1 Department of Nutrition, Faculty of Health Sciences, Campus Universitário Darcy Ribeiro, University of Brasília, Distrito Federal 70910-900, Brazil; vcginani@gmail.com (V.C.G.); raquelbabotelho@gmail.com (R.B.A.B.); wilma.araujo@terra.com.br (W.M.C.A.); 2 Department of Statistics, University of Brasilia, Brasilia 70910-900, Brazil; eynakano@gmail.com

**Keywords:** eating competence, validation, questionnaire, psychometrics

## Abstract

This study aimed to translate and validate the Satter Eating Competence Inventory (ecSI2.0^TM^) from English to Brazilian Portuguese. The process included three steps: (i) translation and back-translation of the original ecSI2.0^TM^ to Brazilian Portuguese; (ii) evaluation of its reproducibility; (iii) a pilot study to validate the Brazilian version of the Satter Eating Competence Inventory (ecSI2.0^TM^BR) for a selected sample of the Brazilian adult population (internal consistency and factor validity). The reproducibility (test–retest reliability) was verified using the intraclass correlation coefficient (ICC) obtained by the responses of 32 Brazilian adults. All domains of the ecSI2.0^TM^BR and the total score showed ICC > 0.8. Considering the entire questionnaire, none of the domains presented significant divergences among the participants’ responses (*p* < 0.001). In the pilot study with 662 individuals, 74.9% (*n*= 496) were female, mean age was 40.33 ± 12.55, and they presented a higher level of schooling and income. Analyses revealed Cronbach’s alpha coefficients of 0.869 for the ecSI2.0^TM^BR total scale, 0.793 for Eating Attitudes, 0.527 for Internal Regulation, 0.728 for Food Acceptance, and 0.822 for Contextual Skills. In general, the ecSI2.0™BR presented good acceptability, showing total floor and ceiling effects of ≤0.6%. Factor validity was examined by confirmatory factor analysis. The four domains presented a good fit in the confirmatory factor analysis: RMSEA = 0.0123 (95% CI: 0–0.0266); CFI = 0.998; χ^2^ = 75.9; df = 69; *p* = 0.266. The ecSI2.0^TM^BR is the first tool designed to measure eating competence (EC) in the Brazilian population, showing good reproducibility and internal consistency. We expect the ecSI2.0^TM^BR will support innovative research to investigate the association of EC and health outcomes, as well as new strategies based on emerging behavioral theories to enhance nutritional education policy.

## 1. Introduction

Eating is a complex process composed of learned behavior, social expectations, acquired tastes, attitudes, and feelings about eating and/or about a food item [1]. Eating competence (EC) is described as an attitudinal and behavioral concept that has been associated with higher diet quality and better nutrition [2,3,4]. EC has been increasingly studied in some countries since competent eaters tend to have higher dietary quality [2,3], more excellent parent modeling of healthful eating behaviors [5,6], and are physically more active [7]. Additionally, they tend to have more positive attitudes and behaviors toward food and eating [8,9] and less stress [10], as well as lower blood pressure [11], greater weight satisfaction [9], and less overweight/obesity [12,13]. Furthermore, EC is associated with better sleep quality [14,15,16], less disordered eating [12,13,17], and less emotional and uncontrolled eating [12,13]. Recent findings suggest that improving eating competence, especially for planning and having regular meals, could promote adherence to a healthy diet and, in the long term, also support the prevention of type 2 diabetes [18].

In this sense, the Satter Eating Competence Model (ecSatter) was proposed as a biopsychosocial model of eating behavior considering four components: Eating Attitudes—having relaxed enjoyment of preferred foods in satisfying amounts; Food Acceptance—being interested in food, experimenting with unfamiliar food, and achieving dietary variety based on food enjoyment and learned food preferences; Internal Regulation—being naturally attentive to internal signals of hunger, appetite, and satiety to guide how much to eat, supporting stable body weight; and Contextual Skills—having resources to manage the food context, planning and providing regular and reliable opportunities to eat [1,4]. EC is the result of the successful adoption of these four components [13]. It is a behavioral model not focused on nutrients, portion size, or food groups. Instead, it focuses on enjoying food and eating, paying attention to variety in the diet, attending to signals of hunger and satiety, and preparing meals and snacks regularly with some attention to nourishing food and the environment in which it is consumed [1,19]. The increasing prevalence and severity of diseases related to food and nutrition point to the need for behavioral approaches to understand food choices [20]. In this context, measuring the components of EC is relevant in order to promote nutritional health.

Therefore, the Satter Eating Competence Inventory (ecSI2.0^TM^) instrument evaluates EC using the interrelated spectrum of eating attitudes and behaviors. The instrument is composed of a 16-question tool designed to assess the four components of EC. This tool allows researchers and educators to follow intervention outcomes and to explore the eating competence construct [21]. It is validated for English use and has been translated and approved in Arabic, German, Japanese, Finnish, and Spanish [21].

However, there is no study using this tool in developing countries, and the ecSI2.0™ has not been validated for Latin American countries, like Brazil. Poor diet quality, overweight, and other chronic diseases have been increasing among Brazilian adults [22], and little has been achieved to improve healthy eating habits in most of the Brazilian population despite the knowledge about the relationship between food and health. New findings of eating behavior could be useful to trace health promotion policies targeted to the Brazilian population.

Therefore, the present study aimed to translate and validate the ecSI2.0^TM^ from English to Brazilian Portuguese. We expect that this new tool will allow future research on EC among the Brazilian population. Potentially, this could also help health professionals and governmental institutions to develop strategies and public policies concerning diet, nutrition, and health.

## 2. Materials and Methods

This cross-sectional study, approved by the Ethics Committee of Santa Marta’s Institute of Teaching and Research, Federal District, Brazil (CAAE 24415819.2.0000.8101), was performed in three steps: (i) translation and back-translation of the original ecSI2.0^TM^ to Brazilian Portuguese; (ii) evaluation of reproducibility; (iii) a pilot study to validate the questionnaire for the Brazilian adult population (internal consistency and factor validity).

### 2.1. Translation and Back-Translation of the ecSI2.0™

Translating an existing research instrument into another language is more convenient than developing a new instrument and allows comparisons among people from different countries [23]. The quality of the translation affects the validity of the results, and the adaptation process has to consider linguistic and cultural differences [24].

The original ecSI2.0^TM^ inventory consists of 16 items scored on a five-point Likert scale and assigns values as following: Always = 3; Often = 2; Sometimes = 1, Rarely = 0; Never = 0. The score is defined as the sum of the responses for each of these items. Thus, ecSI2.0^TM^ can take values from 0 to 48 [21]. Competent eaters have a minimum total score of 32 [12,21], and higher values in the ecSI2.0^TM^ indicate greater EC. This index has four domains: Eating Attitude, composed of six items (“I am relaxed about eating”; “I am comfortable about eating enough”; “I feel it is okay to eat food that I like”; “I am comfortable with my enjoyment of food and eating”; “I trust myself to eat enough for me”; and ”I enjoy food and eating); Food Acceptance, with three items (“I experiment with new food and learn to like it”; “If the situation demands, I can “make do” by eating food I don’t much care for”; and “I eat a wide variety of foods”); Internal Regulation, consisting of two items (“I eat as much as I am hungry for” and “I eat until I feel satisfied”); and Contextual Skills, with five items (“I have regular meals”; “I tune in to food and pay attention to eating”; “I make time to eat”; “I consider what is good for me when I eat”; and “I plan for feeding myself”) [19,21]. There are no established score cutoffs for each of the subscales [21].

In this step, the ecSI2.0^TM^ inventory was translated to Brazilian Portuguese according to the translation process indicated by the NEEDs Center [25]. Two native Brazilian Portuguese speakers, who had not previously been exposed to the ecSI2.0^TM^, translated the instrument from English into Brazilian Portuguese. A researcher, familiar with the Satter Eating Competence Model, discussed with the two translators the two versions and reached a single version. Five native speakers, who were naïve to the subject, pretested the preliminary Brazilian Portuguese version. Each of the test-takers was asked to express, question by question, their understanding of the meaning of each item. Based on the test-taker responses, the translators came up with a single preliminary Brazilian Portuguese version. A native Brazilian Portuguese speaker, who was not involved in the previous steps, performed the back-translation from the Brazilian Portuguese version into English. Researchers sent the translation table (with translation, back-translation, and comments) to the NEEDs Center [21] for the agreement assessment of the intent and meaning of the items. NEEDs Center researchers suggested some changes that were discussed and evaluated by the Brazilian researchers and the translators, to reach the final ecSI2.0^TM^BR by group consensus. The use of translation, in conjunction with back-translation, including bilingual and monolingual participants, is the recommended method to maintain the idea of each item in another language [23].

### 2.2. Evaluation of Reproducibility of the ecSI2.0^TM^BR

The reproducibility of the ecSI2.0^TM^BR was analyzed before a more extensive application. It was assessed for test–retest reliability, as this shows the ability to produce consistent results when used multiple times under nearly similar conditions [26,27]. According to Zou (2012) [28], the sample required for a hypothesized (based on previous data or experience) intraclass correlation coefficient (ICC) of 0.85 to be statistically greater than 0.6, considering a significance level of 5% and power of 80%, is 21 observations. For this purpose, on November 30, 2019, considering that not all of the individuals invited would answer the questionnaire, a convenience sample of 50 Brazilian individuals received an invitation through email, messaging apps, and social networks to answer the questionnaire to achieve a minimum of 21 individuals participating in the test–retest. The inclusion criteria were to be Brazilian, over 18 years old, and answer the entire questionnaire. From this sample, 36 met the criteria and answered the questionnaire entirely. To evaluate the reproducibility (test–retest reliability) of the ecSI2.0™BR, the questionnaire was sent again after 24 h, and each of the 36 respondents was asked to answer it. The participants did not previously know about the need for a second response. From the 36 invited participants, 32 accepted to participate again, and the reproducibility was performed using the responses of these 32 Brazilian adults (56.2% were female, mean age was 41.21 ± 10.74 years old, and 96.9% had a minimum undergraduate schooling level). The mean interval between the two responses was 58.6 ± 43.2 h, as not all participants responded as soon as they were asked.

The questionnaire was answered using the SurveyMonkey^®^ tool, an online survey platform. The reproducibility of the ecSI2.0^TM^BR was verified by its total score and by domains through the intraclass correlation coefficient (ICC) with two random effect models and absolute agreement definition. The analysis was based on a single measure, and values equal to or greater than 0.6 indicated that the instrument had a good level of reproducibility [26].

### 2.3. A Pilot Study to Validate the ecSI2.0^TM^BR (Evaluation of Internal Consistency)

The final ecSI2.0^TM^BR was validated by a pilot study with a convenience sample of the adult population (20–59 years old) living in the Federal District, Brazil. According to Hair et al. [29], the process of validating a questionnaire requires 20 respondents per item (20:1). In this sense, the minimum sample size was estimated to be 320 participants to validate a questionnaire composed of 16 items. The invitation to participate was sent through email, messaging apps, and social networks. Participants received a consent form approved by the Research Ethics Committee, and those who accepted, met the inclusion criteria (age ≥ 18 years), and answered the entire questionnaire were included in the pilot study. The SurveyMonkey^®^ tool was used for this step. With the data of this pilot study, the internal consistency of the instrument and its domains were verified using the Cronbach’s alpha measure. Values equal to or greater than 0.7 were considered consistent [30].

In addition to the ecSI2.0^TM^BR, socio-demographic data (gender, age, income, schooling level, and housing area) were collected using the questions from the Brazilian National Institute of Geography and Statistics (IBGE) [31] as a reference to characterize the studied population.

### 2.4. Statistical Analysis

The questionnaire’s responsiveness was verified by the floor and ceiling effects. The floor effect is observed when the ecSI2.0^TM^BR (and its domains) produces a score equal to zero. The ceiling effect occurs when the instrument (and its domains) reaches maximum values. The factor validity was verified by confirmatory factor analysis. The chi-squared test of minimum discrepancy (χ^2^), the root mean square error of approximation (RMSEA), and the comparative fit index (CFI) evaluated the factor validity [24]. Both RMSEA and CFI range from 0 to 1 (RMSEA = 0 and CFI = 1 indicate a perfect fit). The model fit is accepted when RMSEA is less than or equal to 0.05 [32], and CFI is greater than or equal to 0.9. The scores of the eating competence index were described in terms of means and standard deviation (SD). All tests were performed considering bilateral hypotheses and a 5% significance level. The analyses were performed using IBM SPSS (IBM SPSS Statistics for Windows, IBM Corp, Armonk, NY, USA) and IBM SPSS Analysis of Moment Structures (AMOS) version 22 (Amos, IBM SPSS, Chicago, IL, USA).

## 3. Results

**Figure 1:**
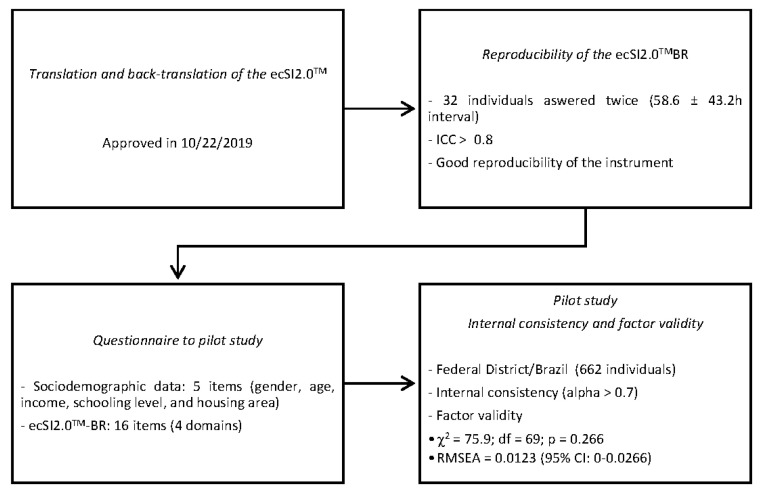
Flowchart of the steps to translate and validate the Satter Eating Competence Inventory in Brazilian-Portuguese (ecSI2.0^TM^BR).

After four judgment rounds, the ecSI 2.0^TM^BR was successfully approved by the NEEDs Center on 10/22/2019 with its 16 items. Figure 1 describes the steps of the translation and validation of the ecSI2.0^TM^BR.

The reproducibility (test–retest reliability) was verified using the intraclass correlation coefficient (ICC). All domains of the ecSI2.0^TM^BR and the total score are shown in Table 1. Analyses revealed that ICC = 0.931 for the ecSI2.0™BR total scale, 0.927 for Eating Attitudes, 0.809 for Food Acceptance, 0.876 for Internal Regulation, and 0.946 for Contextual Skills.

### A Pilot Study to Validate the ecSI2.0^TM^BR for the Brazilian Adult Population

The questionnaire with socio-demographic data and the ecSI2.0^TM^BR were available online from December 2019 to February 2020 using the SurveyMonkey^®^ platform. On the platform, only one answer per device was allowed to avoid duplicates and to make sure that respondents were unique completers. From the 678 individuals who responded to the online questionnaire, the final sample was composed of 662 participants, since some participants (*n* = 16) did not provide all the data necessary for their inclusion in the survey. Table 2 shows the socio-demographic characteristics of the individuals who participated in the pilot study. Most of the participants were female (*n*= 496, 74.9%), had a good distribution across the age groups (mean age 40.33 ± 12.55), and presented a higher level of schooling and income.

Table 3 shows data from the internal consistency evaluation. Analyses revealed that Cronbach’s alpha coefficient = 0.869 for the ecSI2.0^TM^BR total scale, 0.793 for Eating Attitudes, 0.728 for Food Acceptance, 0.527 for Internal Regulation, and 0.822 for Contextual Skills.

In addition, the factor validity was examined by confirmatory factor analysis. Table 4 presents the standardized regression weights (factor loadings) between each domain and individual items of the ecSI2.0 ^TM^BR.

## 4. Discussion

This study translated and validated the Satter Eating Competence Inventory (ecSI2.0^TM^) from English to Brazilian Portuguese. In a Latin American country, the present study is the first to perform the validation of an instrument to measure EC using Brazilian Portuguese. The process included the translation and back-translation of the original ecSI2.0^TM^ to Brazilian Portuguese, the evaluation of its reproducibility, and a pilot study to validate the Brazilian version using internal consistency and factor validity. The results indicated an acceptable model fit, and all four components of the ecSI2.0 were correlated with each other and had a high internal consistency. Although the ecSI2.0^TM^ translation has been approved for other languages, it is only validated for English and Finnish use [21]. To enable the use of this instrument in Brazilian Portuguese, the linguistic validation process is recommended because the original instrument was developed in a language other than the target population language [24,33]. Therefore, the first step of this study was to translate/retranslate the ecSI2.0^TM^. Back-translation is commonly used to check the accuracy of translation in cross-cultural studies [23]. Tilles-Tirkkonen et al. [34] explored the utility of a preliminary Finnish translation of the ecSI2.0^TM^ for evaluating EC in adolescents. They pointed out that an approved back-translation was needed to confirm the validity of their findings [34]. The present translation of the ecSI2.0^TM^ followed the instructions of the NEEDs Center [25]. Thus, the ecSI2.0^TM^BR was approved by the NEEDs Center [25], and it is in agreement with the original ecSI2.0^TM^.

The reproducibility analysis was performed using test–retest reliability to assure the capacity to reproduce the results. The sample was higher than the minimum required (*n* ≥ 21). Participants were 32 Brazilian adults (56.2% female), with a mean age of 41.21 ± 10.74 and a good level of schooling and income, who answered the questionnaire at two different times. The mean interval between the two responses was 58.6 ± 43.2 h because respondents took from 24 h to 15 days to answer the second request. The interval from a minimum of 24 h to a maximum of 15 days was selected to minimize the effect of possible confounding variables, such as increasing or decreasing eating competence, which could affect the data. This time interval can be considered sufficient for the reproducibility analysis because it is not expected that eating competence would change over this period. According to McIntire and Miller [27], for test–retest reliability, the interval between the two responses may vary from a few hours to up to several years depending on the construct being measured, on the stability of the construct over time, and on the target population. Additionally, as the interval lengthens, test–retest reliability declines because the number of opportunities for the participants or situation to change increases over time. All domains of the ecSI2.0™BR and the total score showed an ICC > 0.8 (Table 2). None of the items composing the final instrument showed significant divergence among the 32 evaluators (*p* < 0.001), indicating excellent reproducibility [35].

After this stage, a pilot study was performed with 662 adults living in the Federal District, Brazil (74.9% female), with a mean age of 40.33 ± 12.55. This sample size population was twice as large as the minimum sample required to validate a tool composed of 16 items. The good internal consistency of the ecSI2.0BR^TM^ was confirmed, as the Cronbach’s alpha coefficient for the total score was 0.869 (Table 3). In general, the ecSI2.0™BR presented good acceptability [36], showing total floor and ceiling effects of ≤0.6% (Table 3). In the confirmatory factor analysis, the four domains presented a good fit, and all items showed factor loadings > 0.6 and were not considered for removal [29].

Except for the internal regulation domain, which presented an alpha of 0.527, the other items of the questionnaire showed an alpha of >0.7, indicating good reliable internal consistency (Table 3). These results are in line with Stotts and Lohse (2007) [37], who also found a Cronbach’s alpha coefficient score of <0.7 for internal regulation when performing a study to assess the test–retest reliability of the ecSI^TM^ to determine its usefulness as a measure of EC. At that time, the Internal Regulation construct was composed of three statements, and the authors suggested further development and evaluation of the internal regulation subscale [37]. Considering that the Cronbach´s alpha value is affected by the number of items, the low value found in the present study may be explained by the small scale [30]. In 2019, Godleski et al. performed a confirmatory factor analysis to explore the construct validity of the ecSI2.0^TM^ using data from three heterogeneous socioeconomic samples. After that, the ecSI2.0^TM^ was restructured, and the item “I trust myself to eat enough for me” moved from Internal Regulation to the Eating Attitudes subscale. The internal regulation domain is now composed of only two statements [19], which may also have affected the low Cronbach’s alpha value found for the internal Regulation domain [30]. This recent reformulation supports the EC construct by categorizing the 16 items into four subscales: Eating Attitudes (six items), Food Acceptance (three items), Internal Regulation (two items), and Contextual Skills (five items). The authors encourage exploring the performance of the ecSI2.0^TM^ in linguistically diverse populations with confirmatory factor analysis [19], as it was done in the present study using Brazilian Portuguese. The four domains of the ecSI2.0-BR^TM^ showed a good fit in the confirmatory factor analysis, which means that the measures of the constructs are consistent with the understanding of their nature.

A limitation of this study might be the online application of a self-administered instrument. This method was chosen because it is less costly and less invasive. It requires less effort, and less time for the researchers and the participants, than a face-to-face interview, which can also cause difficulties in reaching part of a population, due to geographical limitations [38]. A study comparing online and paper–pencil formats found that the ecSI^TM^ scores did not differ according to the method of survey completion [12]. Data from the Brazilian population (2017) show that three out of four Brazilians have internet access, and the level of cellphone possession, a primary tool used to access the internet, increased from 92.6% to 93.2% [39]. Therefore, the online self-reported method shows efficiency for data collection, the possibility to reach a more significant number of participants, and a positive impact on cost.

Another limitation of the present study might be the fact that our sample had a higher proportion of female (74.9%) respondents. However, in Brazil, females play a central role in family food purchasing and preparation, reporting sole responsibility for household food decisions [31,40]. Moreover, the first study designed to validate the ecSI^TM^ [12] also presented a predominantly female sample (78.7%). In general, women tend to be more concerned about their health and participate in health surveys more than men [33,41]. Thus, this high proportion of females could have resulted in a higher validity. Nevertheless, the sample was larger than the minimum required for the validation process, thereby making the study findings remarkable.

There was also a selection bias regarding the educational and socioeconomic level of the sample, as they were mostly graduated and presented high income. The ability to eat competently may be affected by food insecurity and/or income restrictions. For example, Lohse et al. (2007) reported that “worrying about money for food” and “running out of food by the end of the month” were associated with a lack of eating competence [12]. Krall and Lohse [13], after noticing that lower income persons tend to show lower EC, performed a study with 507 low-income women, aged 18 to 45 years, to investigate their interpretation of the meaning of the ecSI™ and its validation with a lower income audience. Although the ecSI™ for low-income people was shown to be valid in measuring EC for persons with a higher socioeconomic position [42], this relationship needs to be tested among the socioeconomically disadvantaged Brazilian population as well. According to a census by the Brazilian Institute of Geography and Statistics (IBGE), approximately half of the Brazilian population is 25 years of age or younger and has less than an eighth-grade education [31]. The mean age of the pilot study sample was 40.33 ± 12.55, and 53% of the respondents had a graduate degree. In the pilot study, participants were more educated than the Brazilian population in general, and the mean age was rather high, which has been associated with higher eating competence [2,12], so the results cannot be generalized to young people and persons of a lower socioeconomic position. Therefore, further studies are necessary to evaluate EC in these populations.

## 5. Conclusions

The Brazilian Portuguese version of the ecSI2.0^TM^ was the first tool designed to measure EC in the Brazilian population. Validation is a continuing and ongoing process [26], and in this study, the ecSI2.0™BR showed good reproducibility, internal consistency, and a good fit in the confirmatory factor analysis. It represents the first tool designed in Brazilian Portuguese to investigate EC among Brazilian adults. We expect the ecSI2.0™BR will support innovative research to investigate the association of EC and health outcomes, as well as new strategies based on emerging behavioral theories to enhance nutritional education policy.

## Figures and Tables

**Table 1:** Reproducibility of the ecSI2.0^TM^BR domains (*n* = 32 participants).

ecSI2.0 Domains	ICC ^a^ (*p*)	95% CI
Eating Attitudes	0.927 (<0.001)	0.850–0.965
Food Acceptance	0.809 (<0.001)	0.658–0.872
Internal Regulation	0.876 (<0.001)	0.749–0.939
Contextual Skills	0.946 (<0.001)	0.889–0.974
Total	0.931 (<0.001)	0.857–0.967

^a^ Interclass correlation coefficient (ICC).

**Table 2:** Socio-demographic characteristics of the individuals (*n* = 662, Federal District, Brazil).

		Sample (*n* = 662)	
		Freq	%
**Gender**	Female	496	74.9%
	Male	166	25.1%
**Age**	Up to 30 years	169	25.5%
	31 to 40 years	146	22.1%
	41 to 50 years	184	27.8%
	51 to 59 years	163	24.6%
**Schooling Level**	High School	55	8.3%
	Undergraduate	256	38.7%
	Graduate	351	53.0%
Income *	Up to 3000 BRL	82	12.4%
	3001 to 5000 BRL	62	9.4%
	5001 to 10,000 BRL	130	19.6%
	10,001 to 20,000 BRL	201	30.4%
	More than 20,000 BRL	187	28.2%

* BRL: Brazilian Real is the official currency of Brazil and 1.00 USD = 5.56 BRL (5 May 2020).

**Table 3:** The ecSI2.0^TM^BR scores, responsiveness, and internal consistency of the questionnaire.

	Mean (SD)	Median (IQR *)	Range	Floor Effect (%)	Ceiling Effect (%)	Cronbach’s Alpha
Eating Attitudes	11.90 (3.87)	12 (9–15)	0–18	0.3%	6.0%	0.793
Food Acceptance	5.11 (2.44)	5 (3–7)	0–9	3.9%	9.1%	0.728
Internal Regulation	3.76 (1.53)	4 (3–5)	0–6	3.2%	15.1%	0.527
Contextual Skills	9.26 (3.70)	10 (7–12)	0–15	1.2%	6.5%	0.822
Total	30.03 (8.85)	31 (24.37)	0–48	0.2%	0.6%	0.869

* IQR: Interquartile range.

**Table 4:** Standardized regression weights between domains and individual items of the ecSI2.0 ^TM^BR.

Domain	Item	Factor Loadings
Eating Attitudes	I am relaxed about eating	0.7246
	I am comfortable about eating enough	0.7149
	I enjoy food and eating	0.6569
	I am comfortable with my enjoyment of food and eating	0.6763
	I trust myself to eat enough for me	0.6463
	I feel it is okay to eat food that I like	0.7562
Food Acceptance	I experiment with new food and learn to like it	0.6926
	If situations demand I can “make do” by eating food I don’t much care for	0.7280
	I eat a wide variety of food	0.7730
Internal Regulation	I eat as much as I am hungry for	0.6134
	I eat until I feel satisfied	0.6062
Contextual Skills	I tune to food and pay attention to eating	0.8987
	I make time to eat	0.7725
	I have regular meals	0.7451
	I consider what is good for me when I eat	0.8947
	I plan for feeding myself	0.7198
